# Supplementary material for: Two-dimensional video-based analysis of human gait using pose estimation
Source: PLoS Comput Biol. 2021 Apr 23;17(4):e1008935. doi: 10.1371/journal.pcbi.1008935 (PMC8099131; doi:10.1371/journal.pcbi.1008935)
Supplement: Attachment — Submitted filename: Response to Referees 2021.02.08.docx [file pcbi.1008935.s002.docx]

**Re: PCOMPBIOL-D-20-01556 "TWO-DIMENSIONAL VIDEO-BASED ANALYSIS OF HUMAN GAIT USING POSE ESTIMATION"**

**REVIEWING EDITOR COMMENTS:

Thank you very much for submitting your manuscript "Two-dimensional video-based analysis of human gait using pose estimation" for consideration at PLOS Computational Biology.**

**As with all papers reviewed by the journal, your manuscript was reviewed by members of the editorial board and by several independent reviewers. In light of the reviews (below this email), we would like to invite the resubmission of a significantly-revised version that takes into account the reviewers' comments.**

**The reviewers raised several important points. Specifically, please address the applicability of the method. How likely/ready the method is for a clinical application? Is it just proof of concept? In addition, the reviewers are concerned regarding the accuracy measures. Is it possible to quantify the accuracy?**

**We cannot make any decision about publication until we have seen the revised manuscript and your response to the reviewers' comments. Your revised manuscript is also likely to be sent to reviewers for further evaluation.**

We thank the editor for the opportunity of revise the manuscript. In response to the reviewer comments we have made the following major changes to the manuscript:

1. Clarified the applicability of our approach.
2. Rewritten the Abstract and Results to emphasize quantitative description of the results.
3. Added new metrics (including absolute error) to provide more detail about the accuracy of our approach.
4. Added Discussion paragraph to provide context to the accuracy of results by comparing performance to relevant biomechanics literature.
5. Added paragraph to discuss the applicability and generalizability of the current approach for other (clinical) settings.

Please find below our responses to each comment (numbered and restated in **bold**). In some cases, excerpts from the revised text is included in the responses (in *italics*).

**-----------------

REFEREE COMMENTS:

Referee #1:

Major Comments:**

**This study investigates the accuracy of vision-based motion analysis in enabling extraction of biomechanically and clinically relevant outcomes, such as spatiotemporal and kinematic parameters of gait. Given the increasing popularity of vision-based techniques, and their potential to enable deployment of lightweight gait analysis tools across clinics and patient homes, this study is relevant and timely. However, the manuscript is crippled by weaknesses in presentation, technical rigor, and interpretation.**

**The major contribution of the work needs to be clarified: does the work offer a technical or an experimental contribution, or both? The authors seem to suggest both, but do not make a strong case for either. For the experimental component: what were the hypotheses? If not hypothesis-driven (more discovery-driven), could it be stated up front what the authors expected to find and why? For the technical contribution: it is stated that a framework that is built on top of a computer vision algorithm (OpenPose) will enable individuals with no programming background (e.g., clinicians) to use OpenPose. This framework is still provided in Matlab to my understanding and is not generalizable to other datasets without further programing. Will the provided tool generalize to other data collection configurations as is? If the contribution is to enable non-programmers, then further experiments on the generalizability of the provided tools need to be provided. How does the tool perform on data collected elsewhere?**

We thank the reviewer for their detailed and thoughtful comments which have been helpful to improve the revised manuscript.

This study was intended to be an important first step toward understanding the potential for using state-of-the-art human pose estimation algorithms (e.g., OpenPose) to perform quantitative, full-body gait analysis. Interest in using these pose estimation algorithms to track movements in humans and other animals has exploded recently in the behavioral neuroscience and computer vision communities, but there is very little information regarding the accuracy with which these algorithms can track and quantify commonly studied and clinically relevant human movement tasks like walking. Given the lack of apples-to-apples data comparing pose estimation outputs to ground-truth motion capture data during overground walking trials, our manuscript aimed to inform the reader about the degree of accuracy that is possible when using OpenPose for gait analysis. We consider the validation shown here to be a necessary and promising initial step toward the development of a versatile, generalizable approach that could be used flexibly across many settings (including the home and clinic).

We agree with the reviewer that additional work is required to test (and likely improve) the versatility of this approach. There is a need to understand how well pose estimation algorithms can track human gait in different locations, from different viewpoints, and in different patient populations. Our group is currently working on projects designed to address these issues. However, we do not anticipate that additional programming would be required. Our software provides a scaling tool that can be used to convert pixels to actual distances in any location as long as there is an item or markings of known distance within the field of view and along the walking axis. This scaling tool was included specifically to facilitate generalizability of the approach. Furthermore, calculations of temporal metrics and metrics that rely on relative vs. absolute spatial coordinates (e.g., joint angles) are unaffected by the data collection configuration (as long as a sagittal view of the participant is recorded).

The post-processing workflow is indeed provided in MATLAB. We are currently in the process of translating the workflow into other programming languages. Given the current limitations on human subjects research due to the pandemic, we aimed to get the workflow out to users as soon as possible so that they could use it themselves, build upon it, and/or test it in their own data collection spaces. We have now included a discussion paragraph on user generality.

**Overall the manuscript provides no interpretation of whether the accuracy of the extracted parameters is sufficient for specific applications in clinical biomechanics. The language is descriptive and imprecise, referring to results as “reasonably accurate,” for example. What may be reasonable to one, might not be to another – scientist, clinician, field. Also, differences of 0.001±0.023 seconds are reported, but this is below the time resolution with which the data were captured.**

We have revised the wording in the manuscript to lessen subjectivity. We have also added a paragraph to the Discussion that puts the results into context with the biomechanics literature, and we now present and discuss temporal parameters in relation to the temporal resolution with which these were acquired in order to put them into context of the expected precision.

**Minor Comments:**

1. **Line 23: “immobile” in what sense?**

We refer to three-dimensional motion capture systems as “immobile” because these are used in a designated laboratory space and are not readily moved to other locations where it would be useful to obtain a gait analysis.

2. **Lines 32-36: “well”, “less accurate”, and “improved agreement” are qualitative descriptions and the abstract should include quantitative findings backed up by statistics (no need to include statistical tests in the abstract)**

We have rewritten this part of the abstract to emphasize a quantitative description of the data (page 2, lines 32–39):

*“When assessing all individual steps in the walking bouts, we observed mean absolute errors between motion capture and OpenPose of 0.02 s for temporal gait parameters (i.e., step time, stance time, swing time and double support time) and 0.049 m for step lengths. Accuracy improved when spatiotemporal gait parameters were calculated as individual participant mean values: mean absolute error was 0.01 s for temporal gait parameters and 0.018 m for step lengths. The greatest difference in gait speed between motion capture and OpenPose was less than 0.10 m s^−1^. Mean absolute error of sagittal plane hip, knee and ankle angles between motion capture and OpenPose were 4.0°, 5.6° and 7.4°.”*

3. **Line 61: “use of a large dataset containing walking sequences of many individuals”. What does “large” mean in this context?**

We have removed “large” from the sentence so that it reads (pages 4–5, lines 79–81): *“… use of a dataset containing overground walking sequences with synchronized three-dimensional motion capture and video recordings, including video recordings from multiple perspectives ...”*

4. **Line 66: “an “out of the box” approach with no need for additional network processing.” What kind of “out of the box” approach?**

By an ‘out of the box’ approach we mean that we run OpenPose without any specialized commands (e.g., commands to track only a single person) and that we do not employ other deep learning networks to derive gait parameters (see for example Kidziński et al. (2020) *Nat Comm* **11**). We have omitted the phrase ‘out of the box’ as it may be confusing.

5. **Line 73: 135 keypoints? Could you please clarify what these 135 keypoints correspond to? Joint centers across different people in the same image?**

The 135 keypoints correspond to labeled anatomical locations of a single person identified in a video image. OpenPose offers to track several additional models than what the “BODY_25” model (consisting of 25 keypoints) that we used in the current study: there are a models of the hands, feet and face. Combined, these models can track up to 135 keypoints of a single person. We have clarified the sentence to read (page 5, lines 90–92): *“ … human pose estimation algorithm that uses Part Affinity Fields to detect up to 135 keypoints (using models of “body”, “foot”, “hand” and “facial” keypoints) in images of humans … “*

6. **Line 87 and Line 92: What is reasonably accurate? I suggest refraining from using subjective verbiage in a scientific article**

We have removed this statement so that the sentence reads (page 6, lines 104–107): *“We consider that there may be opportunity to improve upon the results presented in the current study by adjusting OpenPose parameters, using other pose estimation algorithms, or using other methods of video recording.”*

7. **Lines 94-100: These are methods, not results.**

Because Articles in PCB are structured with the Methods section at the end, we consider it important to briefly give an overview of the dataset analyzed and the main comparisons of interest (motion capture and OpenPose left and right side views) before the results are presented.

8. **Line 104: Please refer to tables parenthetically.**

We have rewritten much of the Results section and now only refer to tables within parentheses.

9. **Line 109 -111: This is not a result.**

We have omitted this sentence.

10. **Lines 118 – 122: These descriptions belong in the Methods section.**

We have omitted this paragraph as suggested.

11. **Line 132: I suggest leaving “somewhat” out and finding more precise language to describe your findings. Overall, these casual terms can diminish the seriousness with which your science is perceived.**

We appreciate the suggestions to remove subjective description of the results. We have rewritten the Results section to reflect a quantitative description and have removed “somewhat” and related qualitative descriptors.

12. **Lines 159 – 161: “Pearson and intra-class correlation coefficients of step length between OpenPose left (C1) and right (C3) side views were 0.671 for all steps and ≥0.927 for individual participant means.” Statistics are used to back up a finding, and I recommend that they do not become the topic. It is best to state your finding and back it up parenthetically with the appropriate statistic.**

We have rewritten the Results section as recommended.

13. **Line 164: “All correlations were statistically significant.” See above.**

We also revised this section per the recommendations of the reviewer.

14. **Lines 174 – 186: Are these findings specific to the video data you used or can anything more generalizable be learned from them?**

We consider the systematic errors in step length calculation described here to be generalizable to other approaches of using sagittal plane videos of gait because of the parallax and changes in perspective that will inevitably occur when the participant moves closer to the edges of the field of view of the camera.

15. **Lines 187-195: These are not results, but rather discussion points. Also, it seems quite obvious that (1) the quality of OpenPose’s estimation will degrade as the subject moves away from the camera, (2) “step length estimation is influenced by whether step length is measured at left or right heel-strike,” as this relates to the position of the leg versus the camera, and (3) “individual step lengths are estimated most accurately when the person is in the center of the field of view of the camera.” Did the authors not expect these findings?**

We have now moved this paragraph to the Discussion section. We think that it may be helpful to some readers to explain the systematic errors in step length as they may not be obvious to all readers.

16. **Line 242: “Gait events (e.g., heel-strikes and toe-offs) identified by OpenPose matched those identified by motion capture very well (i.e., generally within one motion capture frame).” Can you comment on how this is possible when the video data has much lower temporal resolution?**

Since motion capture data was recorded with a temporal resolution of 10 ms, the time difference between motion capture and OpenPose (assessed with a 40 ms time resolution of video recordings) will be multiples of temporal resolution of the motion capture system.

17. **Lines 490 – 502: Calculation of joint angles is unclear.**

We have expanded on the calculation of sagittal plane joint angles (pages 26–27, lines 565–574):

*“We calculated sagittal plane hip, knee and ankle angles of left and right legs using two-dimensional marker (motion capture) and keypoint (OpenPose) coordinates. The hip joint center in the motion capture data was estimated based on a regression model (45). We used the following markers (motion capture) or keypoints (OpenPose) to calculate joint angles: hip angle was the vector between hip and knee (0° is vertical, flexion is positive, extension is negative); knee angle was the angle formed by the vectors between hip and knee and between knee and ankle (0° is vertical, flexion is positive, extension is negative); ankle angle was the angle formed by the vectors between knee and ankle and between ankle and toe marker or big toe keypoint (for motion capture and OpenPose, respectively) (0° is horizontal, dorsiflexion is positive, plantarflexion is negative).”*

**Referee #2:

Overall I found this to be an interesting paper and very well written. The authors have done a nice job exploring a relevant topic and (for the most part) clearly and transparently presenting their results. The comments embedded in the pdf are mostly minor - though there are quite a few of them.**

**My one major concern is the authors' choice of correlation as a measure of performance in the comparison to kinematics. In fact, the performance in this realm appears quite poor, contrary to the way the authors portray it. Note that this does NOT make the research less valuable - though it does raise doubts about the authors' devotion to an "out-of-the box" solution.**

**My impression is that the authors are a bit overly-optimistic about the technology and its potential application to real world (especially clinical) problems. I urge them to temper this enthusiasm with a bit more realism regarding the relatively poor performance of the method on kinematics.**

We thank the reviewer for the comments which we have used to increase clarity and provide more context to the results.

Following the suggestions of the reviewer, we have omitted the correlations of the peak angles and replace with mean absolute error comparisons. We think this have improved the clarity of the results of gait kinematics.

We have sought to put more emphasis on context of the results which we think will present the accuracy that can be expected by other when using the approach described in this study. We also believe that this approach has toned down the enthusiasm presented in the manuscript. **General comments:**

1. **Line 33: It would be useful to give some idea of "well" and "less accurate". I understand there are lots of parameters estimated - but perhaps choose a few KEY ones.**

We have refocused the Abstract to present quantitative information about the comparisons between motion capture and OpenPose analysis (page 2, lines 32–39):

*“When assessing all individual steps in the walking bouts, we observed mean absolute errors between motion capture and OpenPose of 0.02 s for temporal gait parameters (i.e., step time, stance time, swing time and double support time) and 0.049 m for step lengths. Accuracy improved when spatiotemporal gait parameters were calculated as individual participant mean values: mean absolute error was 0.01 s for temporal gait parameters and 0.018 m for step lengths. The greatest difference in gait speed between motion capture and OpenPose was less than 0.10 m s^−1^. Mean absolute error of sagittal plane hip, knee and ankle angles between motion capture and OpenPose were 4.0°, 5.6° and 7.4°.”*

2. **Line 35: Again - some idea of "improved accuracy further" would be useful. Perhaps percent change (and range of pct change) or something like that.**

As mentioned above, we have revised the abstract to provide more quantitative information about accuracy.

3. **Line 59: This is important - however, the most likely value of this sort of technology is to capture "free living" movement - so claims of gold-standard validation rings a bit hollow. Previous authors have calibrated against gait-lab environment data, which is what this work does as well.**

We appreciate the point made by the reviewer and we have added a paragraph to the Discussion on the constraints imposed by data collection setup and how the results of the current study may not generalize to other setups outside of the laboratory (page 15, lines 312–320):

*“To apply the OpenPose analysis described in the current study, it is important to consider the choice of data collection setup. We used a dataset that contained stationary video camera recordings of sagittal plane views of healthy human gait. It is possible that factors such as camera height and the distance to plane of progression may influence the results. Furthermore, camera angles that deviate from perpendicular will likely affect results. We expect that temporal parameters are most robust to changes in the data collection setup as it is likely that gait events can be reliably detected with a variety of conditions; on the other hand, step lengths and joint angles are likely more susceptible to be affected by the setup condition (e.g., perspective changes from different camera angles).”*

4. **Line 63: This is very valuable for guiding future optimization.**

Indeed. We consider the comparison across left and right side video viewpoints as an important element in the analysis presented in the current study.

5. **Line 85-86: While I understand this opinion, and appreciate it being made explicit here, I disagree, and feel the approach is misguided. While many may use the software this way, your results (and those of others) show that this is (probably) inadequate. This is merely an opinion, offered for the authors' "benefit" - and has no bearing on my scientific assessment of the paper.**

We appreciate the view of the reviewer regarding this. As we also state in the Introduction, we do not claim the analysis undertaken in the current study to be an optimized approach to human gait analysis. But we think that it is important to state that the neural network we used may be modified.

6. **Line 127-128: your use of "but" confuses me. Overestimating stance time and underestimating swing time are the same thing.**

It is correct, as the reviewer points out, that overestimating stance time and underestimating swing time are the same thing. We have rewritten the Results section so that this sentence is omitted.

7. **Line 129-130: This should be the focus, rather than the p-values, which are irrelevant often, and especially in an analysis like this! I agree with your judgment that these types of errors are small. It would be useful to put them into context of test-retest reliability of the 3DGA data. For example, in our lab we have shown that independent assessment of gait events differs by several frames between trained staff processing the data.**

We have rewritten the Results section so that differences in the temporal gait parameters are related to the temporal resolution with which the data was acquired. As a result, the Results section is not focused on *P* values.

8. **Line 174-186: Issues like this - well described here - indicate why "out of the box" use is unlikely to be a good solution. Do the authors have an idea why this bias exists? It doesn't behave like simple parallax.**

We consider that the perspective created by the depth-difference between the feet changes as the person moves from the edge to the field of view to the middle. As a result of this, step lengths will appear either shorter or longer as a function of the person’s position in the field of view and whether steps are taken on the left or right leg.

9. **Line 192-195: But in the free-living condition (or even laboratory), participants may not be centered during the video capture.**

As the reviewer points out, it is not guaranteed that averaging step lengths over the walking bout will produce a more accurate measurement. This paragraph now points this out (pages 13–14, lines 273–281):

*“If only averaged step lengths are of interest for each participant - perhaps, for example, as a summary statistic in a clinical assessment - OpenPose estimated step length with higher precision in the current study because the systematic errors in step length that occur due to position along the walkway appear to offset when step lengths are averaged across the entire walking bout. Note that averaging step length across the walking bout may not increase accuracy in other scenarios that are different from the current study: e.g., if the participant walks on a treadmill that is not centered in the field of view of the camera, or if the participant walks along an overground walkway but the camera is not centered along the section of the path where the participant is visible.*”

10. **Line 199-201: I disagree with the use of correlation as a measure of performance here. What matters is the error between the two measures. You can have perfect correlation with huge bias, or even high correlation with large errors. In addition - the variation accounted for would be useful in addition to whatever other error metric is used (hopefully difference instead of correlation). I recommend a re-framing of these results to highlight more direct measures of performance - like mean absolute error. You presented differences for the stride parameters, why not do the same here?**

We have rewritten the Results section comparing lower-limb joint angles to put emphasis on the error (mean absolute error; MAE) between motion capture and OpenPose. However, we have retained the cross-correlation results because they show that the time-varying nature of the joint angles are better preserved in hip and knee angles than ankle angles which we consider further evidence that hip and knee angles are better estimated than ankle angles with OpenPose. We find that MAE for individual strides show similar results as when compared across individual participant mean values and so we do not show these results. Rather, we have added an analysis of the effect of walkway position on joint angles (Fig. 8).

11. **Line 210-211: I will just reiterate my view that correlations are not the best way to assess the performance here. What people want is an accurate, not a well correlated, substitute for instrumented 3DGA!**

We have omitted the analysis of peak joint angles and do therefore not present these correlations.

12. **Line 228: further, not initial. As the authors note, there have been several studies published looking at this topic already (11-17)**

We have omitted “initial”.

13. **Line 241: No! Authors showed some accuracy and other measures of correlation.**

We have rewritten this part of the Discussion to give a more detailed summary of the Results and put them into context with other literature.

14. **Line 253-255: I disagree that this is what the findings show. I think the authors should significantly weaken this statement. Performance was clearly good for some things, and bad for others. Good in the mean, bad individually. Good when patient centered, bad when off axis. Etc... This does not take away from the value this work adds - but the authors over-selling of the results (e.g. focus on correlations rather than errors) is disappointing.**

We addressed this point in similar manner to the previous comment.

15. **Line 260: This is a valuable thing to test, but the data shows that, while there is good L/R agreement, the angle of view (walking towards or away from the center) matters. These limitations should be made more clear in this discussion of viewpoints.**

We have put a paragraph on step length calculation in the discussion that more directly addresses the large step length errors observed in the current study (pages 13–14, lines 267–286):

*“From the analysis of differences in step length and anterior-posterior position of the participant along the walkway (Figs. 5 and 6), we identify the following considerations that affect the estimation of individual step lengths with OpenPose: 1) step length estimation is influenced by the position of the participant along the field of view of the camera, 2) step length estimation is influenced by whether step length is measured at left or right heel-strike, and 3) individual step lengths are estimated most accurately when the person is in the center of the field of view of the camera. If only averaged step lengths are of interest for each participant - perhaps, for example, as a summary statistic in a clinical assessment - OpenPose estimated step length with higher precision in the current study because the systematic errors in step length that occur due to position along the walkway appear to offset when step lengths are averaged across the entire walking bout. Note that averaging step length across the walking bout may not increase accuracy in other scenarios that are different from the current study: e.g., if the participant walks on a treadmill that is not centered in the field of view of the camera, or if the participant walks along an overground walkway but the camera is not centered along the section of the path where the participant is visible. When comparing individual participant mean values of step length, the group mean absolute difference between motion capture and OpenPose was less than 0.020 m and the greatest difference was less than 0.060 m. This suggests that OpenPose is capable of step length estimation with the accuracy to detect changes in healthy gait, as these values are less than the minimal detectable change in step length of 0.060 m that have been reported in inter-session, test-retest experiments of healthy human gait (22, 23).”*

16. **Line 285: note that wider angle views are often accompanied by distortion. So, the recommendation for wide angle should be accompanied by "distortion corrected".**

As suggested, we have added the caveat that wider angle views should be accompanied by distortion correction.

17. **Line 290-294: ... or, perhaps knowledge of the participant's height would be enough - they can act as their own ruler.**

This is an excellent point raised by the reviewer (for what it’s worth, we had also considered shoe size). We have now included (pages 17–18, lines 366–370):

*“To generate estimates of gait parameters that incorporate spatial information (e.g., gait speed, step length), it is necessary to scale the video. Here, we accomplished this by scaling the video to known measurements on the ground. This could be done in the home or clinic by placing an object of known size in the field of view at the same depth with which the person is walking or use a known anthropometric quantity such as body height.”*

18. **Line 328: except the errors did not behave like simple parallax, they were negative biased in one direction, and positive in the other.**

We have reworded the sentence to read (page 19, lines 403–406): *“We observed that different perspective toward the edges of the field of view introduced errors into our OpenPose estimates of parameters that rely on spatial information (e.g., step length, Figs. 5 and 6).”*

19. **Line 349-350: This could be a major problem for generalization of results. 1. Small N. 2. "healthy" (by which I assume the authors mean free of significant gait impairments - a terminal cancer diagnosis, in its early stages, will not impair gait). Many previous studies of typical gait have "discovered" things that end up not translating to impairment. This does not invalidate the current study - but the limitation posed by this should be made more apparent.**

As we did not recruit the participants for the current study (we used a freely available dataset) we have described the cohort as “healthy” as in Kwolek et al. (2019) wherein the dataset is described. We have reached out to the original authors and they did not use specific inclusion/exclusion criteria. We have now added a paragraph in the Discussion on how the Results and workflow may generalize to gait pathology (pages 15–16, lines 321–327):

*“The results of the current study were based on healthy human gait – does pathological gait affect how well gait parameters and kinematics are estimated with the described workflow? As an example, factors such as body and limb postures may affect how OpenPose tracks keypoints. If keypoints are tracked different in pathological gait that may potentially have subsequent effects on our workflow and the way in which gait parameters are calculated. We suggest that separate analyses of a range of gait pathologies are needed to establish the accuracy obtained with our workflow.”*

20. **Line 411-415: This step is clearly necessary - but poses big problems for general deployment in free-living spaces. Perhaps some explicit discussion of this limitation (in the "discussion" section) would be useful. In addition to detecting false "people", OpenPose also sees shoes lying around on the ground, shadows, pictures on walls, etc...**

We have added a paragraph to the Discussion section concerning this and other issues related to post-processing (pages 14–15, lines 299–311):

*“Our OpenPose workflow relies on several post-processing steps, some of which were completed manually to clean the data. In about 20% of the video frames analyzed, multiple persons were detected by OpenPose – as only one person was visible in the videos, the additionally detected persons were false positive identifications by OpenPose (note: there is an OpenPose flag available for limiting the maximum number of persons that can be detected in a given frame, should the user be interested in applying it). In this analysis, we manually checked that the person tracked by OpenPose was the participant. Person detection may be an important issue if OpenPose is used to analyze videos in which several individuals are present (e.g., pathological gait in which additional persons are present for safety reasons). We also note that left-right limb identification was switched in about 5% of the analyzed video frames and we manually corrected instances of switched limb identification. The manual portions of the workflow were all based on simple visual inspection of parts of the data and we expect that users with only a basic knowledge of human motion will be able to complete the workflow.”*

21. **Line 425-428: as with the previous issue (false people), this seemingly minor issue can have massive impact when trying this approach "in the wild". Further/explicit "discussion" of this limitation is necessary, especially given the authors' focus on an "out-of-the-box" solution.**

See above.

22. **Line 458-459: another hint that an "out-of-the-box" solution can actually work. Even if the calibration was perfect, in many (?most?) situations where this technology would be desired, such calibration (accurate or not) will be impossible.**

It is true that this sort of calibration is required for the workflow provided in the current study and that this step may limit its applicability.

23. **Line 491-493: This is not a good approximation of the hip center. There are many regression models available in the literature that use these markers (and some anthropometry) to estimate hip centers. The authors should compare this simple hip center model to the accepted gold standards, as it may be an additional source of error.**

We have now estimated hip joint center in the motion capture data by using the regression model described by Bell et al. (1990) *J Biomech* **23** (6).

24. **Figure 2: pretty graph, but (stylistically) I think the choice of point size is way too large. I would think 1/4 the area (and maybe even jittering) would make for a more readable graph. It would be VERY useful (and consistent with my main critique of the analysis - which is a focus on correlations) to add text to each plot indicating the mean absolute error (or RMSE, though MAE is more understandable).**

We have added errors of gait parameters between measurement systems in Tables 2 and 4.

25. **Figure 5: I think this is a fascinating finding. As noted earlier, unless I'm misunderstanding something, it does NOT behave like parallax - which would be symmetric about 0.**

We regard this to be an effect of both parallax (in the different OpenPose views) and the perspective on body limb postures at either left or right heel-strike; together these effects create the systematic effects on step length error.

26. **Figure 7: As noted previously - I feel quite strongly about the use of correlation coefficients as a measure of accuracy. It has been done in some of the references the authors cite - but I still think it's a bad idea. I'm sure the authors understand all the ways signals can be correlated but inaccurate. Furthermore, it is pretty clear that what usually matters is the difference in the magnitude of the estimates from the two systems, not how well they are correlated. The use of correlations hides the nature of the challenges with the o-o-b OpenPose approach.**

We have added mean absolute error comparisons of joint angles between measurement systems as suggested. However, we have kept the correlation coefficient analysis since it demonstrates that OpenPose performs better in preserving the time-varying nature of hip and knee angles than ankle angles.

27. **Figure 8: It will be easy to add text to each figure displaying the MAE (or RMSE).**

We have omitted this figure and the analysis of peak angles.

28. **Figure 9: A graph like this does not mesh perfectly with the authors' summary statement: " in sum, our findings show that OpenPose can provide reasonably accurate estimations of many human gait parameters, with some exceptions"**

We have omitted this figure and the analysis of peak angles.
